# Widely targeted analysis of metabolomic changes of *Cucumis sativus* induced by cucurbit chlorotic yellows virus

**DOI:** 10.1186/s12870-022-03555-3

**Published:** 2022-03-31

**Authors:** Zelong Zhang, Haifang He, Minghui Yan, Chenchen Zhao, Caiyan Lei, Jingjing Li, Fengming Yan

**Affiliations:** College of Plant Protection, Henan Agricultural University, Zhengzhou, 450002 Henan China

**Keywords:** Metabolomics, *Cucumis sativus*, Cucurbit chlorotic yellows virus, Lipids, Flavonoids

## Abstract

**Background:**

Plant metabolites play vital roles in regulating the behavior of herbivore insects. Virus infection can universally alter plant metabolites to manipulate the orientation and feeding behaviors of insect vector, to favor the transmission of virus. Thus, determining the differentially accumulated metabolites of plant upon virus infection could provide insights into understanding how the triple interactions among plant, virus and insect vector happens. Our previous studies have found that vector whitefly *Bemisia tabaci* (Gennadius, Hemiptera: Aleyrodidae) showed different orientation behavior and performance on CCYV-infected and healthy cucumber plants. Cucurbit chlorotic yellows virus (CCYV) is exclusively transmitted by *B. tabaci* in a semi-persistent mode. In this study, we take the CCYV, *B. tabaci* and cucumber as a research system to explore the functions of phyto-metabolites in the triple interactions.

**Results:**

A total of 612 metabolites changed upon CCYV infection were monitored. Metabolites mainly enriched in flavonoids, lipids, nucleotides and their derivatives. At 7 days post CCYV inoculation (dpi), the contents of lipids, terpenoids and flavonoids remarkably decreased, while amino acids, nucleotides and their derivatives notably up-accumulated. At 15 dpi, the accumulation of flavonoids were still significantly reduced upon CCYV infection, while lipids, amino acids, nucleotides and derivatives were remarkably enhanced. Most of significantly increased metabolites were lipids (lysophosphatidylethanolamine, LPE; lysophosphatidylcholine, LPC and their isomers). Also, the number of significantly changed metabolites increased with the infection period. However, only a few organic acids and phenolic acids showed difference between CCYV-infected and healthy cucumber plants.

**Conclusions:**

CCYV infection repressed the defensive flavonoids, terpeneoids metabolism but triggered the lipids, amino acids and nucleotides metabolism with the inoculation period. This result suggests that CCYV-infection makes cucumber plants more susceptible for whiteflies attack and CCYV infection. The reduction of defensive comounds and the increase of amino acids may be partially responsible for enhancing feeding preference of whiteflies to CCYV-infected hosts. CCYV may hijacked lipid metabolism for virus replication and assembly.

**Supplementary Information:**

The online version contains supplementary material available at 10.1186/s12870-022-03555-3.

## Introduction

Plant viral pathogens cause enormous harm to the crop production worldwide annually, thus greatly threatening food security. Transmissions of over 80% of plant viruses depend on insect vectors, such as whitefly, thrips, aphids and planthoppers [1, 2]. The epidemics of viral diseases were always correlated with the feeding behavior and movement of insect vectors between virus infected hosts and healthy hosts. Viruses are a kind of intracellular parasites, so that their life cycle involving replication, protein expression and virion assembly rely on the eukaryotic system of host cells [3, 4], thus resulting in the changes of plants metabolites, like flavonoids, phenols, lipids, alkaloids, etc. [5, 6]. The virus-induced changes in plant physiology directly affect feeding behavior of insect vector and virus spread [7, 8]. It has been revealed that plant virus could manipulate host metabolites to attract insect vectors for feeding [9–11]. Here, we are interested in the changes of plant physiology after CCYV infection. This study would help us further study the CCYV infection mechanism and how CCYV affect insect and plants interactions.

According to the different modes of virus transmission by insect vector, plant virus can be classified into three groups: non-persistent, semi-persistent, persistent viruses [12, 13]. Ways in changing phytochemicals by viruses may vary with transmission modes, and so affecting the development and ingestion behavior of insect vector differently. For example, persistent viruses such as Tomato yellow leaf curl virus (TYLCV) and Tomato spotted wilt virus (TSWV) positively affect growth and development of insect vector [14, 15], while Cucumber mosaic virus (CMV), a well-documented non-persistently transmitted virus, causes a negative effect on the development of its insect vector aphids [16]. Because of the relatively long virus acquisition period, attracting insect vectors continuously feeding on virus-infected host plant is helpful for successful acquisition of persistent viruses. On the contrary, non-persistent viruses are easily lost from inset vector with secreting saliva. Thus, non-persistent viruses negatively affect the feeding behavior of vector by reducing the quality of host plants [13]. Poor quality of host plants would force insect vectors to move to a new host plant and transmit the plant viruses.

Cucurbit chlorotic yellows virus (CCYV, genus Crinivirus, family Closteroviridae) is exclusively transmitted by *Bemisia tabaci* in a semi-persistent mode [17]. It was first discovered in Japan and caused a huge economic loss on melon crops recently in Asia and America [18–20]. Previous studies have showed that *B*. *tabaci* preferred to feed on CCYV-infected cucumber plants compared with on healthy host plants, indicating that CCYV infection regulates the behaviors of vector *B. tabaci* indirectly [21, 22]. However, it remains elusive that specific metabolize pathways contribute to the regulation of vector insects’ behaviors. To explain how CCYV manipulates host plant metabolisms to improve its transmission, we used UHPLC-MS to study the metabolomic changes of host *C. sativus* upon CCYV infection.

It is different from transcriptomics and genomics that metabolomics tells us what’s happening within organisms. Metabolomics links genome to phenome and helps us understand the chemical changes of host plant in a wide vision [23]. It directly shows that how plant respond to viral pathogen infection and how viruses alter host metabolisms to promote virus infection, making plants susceptible for vector insects. Therefore, metabolomics can help us better in understanding the virus transmission and infection mechanisms.

## Results

### Identification of CCYV-infected cucumber seedlings and viruliferous whiteflies

**Fig. 1:**
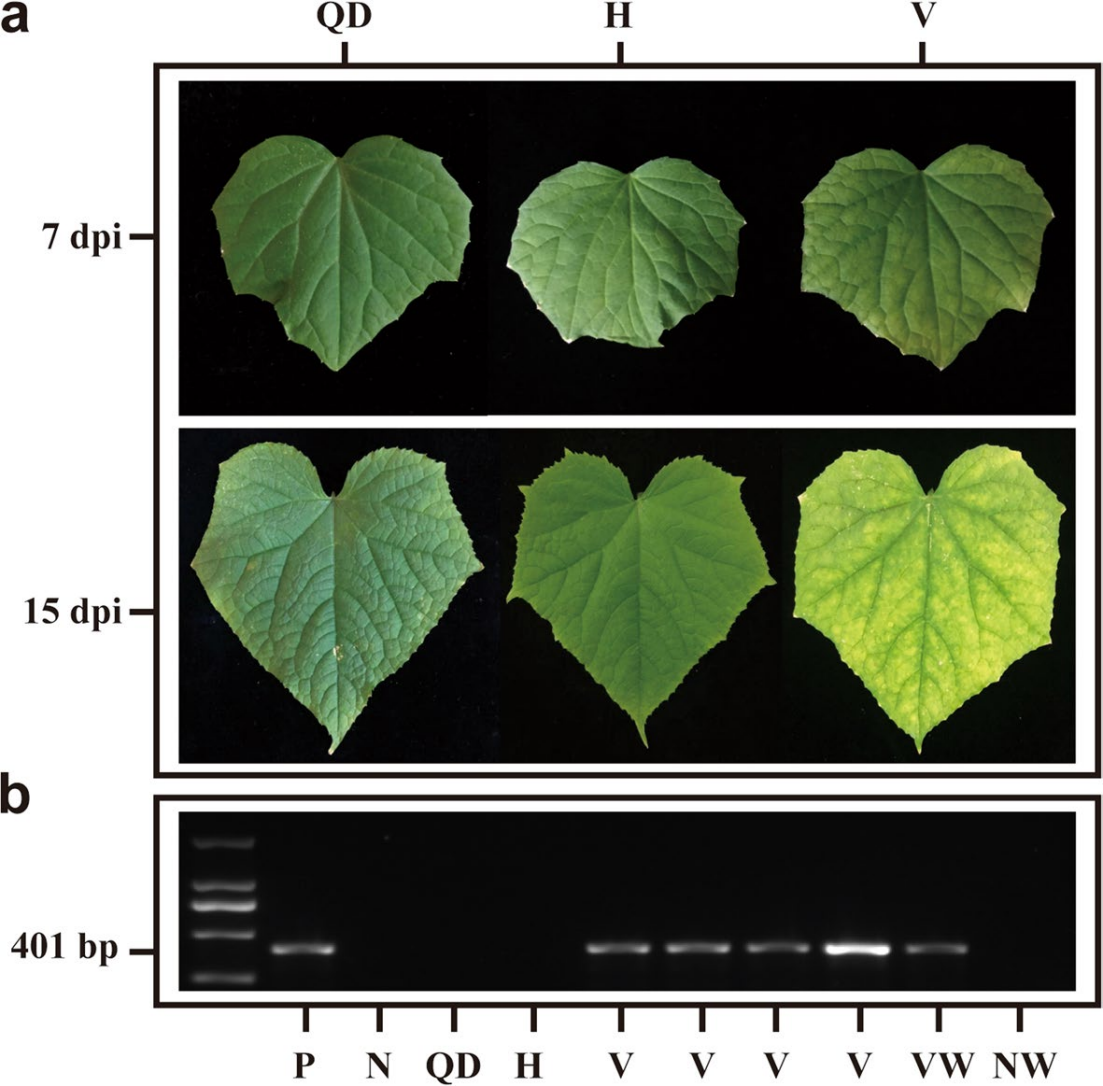
Symptoms (**a**) and identification (**b**) of cucumber plants infected with CCYV QD: Q-biotype damaged plant, H: healthy plants, V: CCYV-infected plants, N: negative control, P: positive control, NW: non-viruliferous whiteflies, VW: viruliferous whiteflies. The full-length gels could be found in figure S1-S2

The CCYV-infected cucumber plants and viruliferous whiteflies were identified by RT-PCR (Fig. 1B). At 7 dpi, the leaves of CCYV-infected cucumber plants show slight yellowing symptoms. However, the yellowing symptoms greatly developed at 15 dpi (Fig. 1A), while leaves of plants in QD and H group remained green.

### Qualification and Quantification analysis of metabolites

The qualitative and quantitative analysis of metabolites were conducted on MetWare database (MWDB), and multiple reaction monitoring (MRM). The widely targeted analysis detected a total of 612 metabolites (Table 1 & Table S1), including lipids, phenolic acids, flavonoids, amino acids, nucleotides and derivatives, organic acids, alkaloids, terpenoids, lignans, coumarins, and tannins. The three most numerous of metabolites are lipids, phenolic acids, and flavonoids.

**Table 1:** Quantity statistics of each class of metabolites

Classes of metabolites	Number of metabolites
Lipids	114
Phenolic acids	91
Flavonoids	89
Amino acids and derivatives	67
Organic acids	61
Nucleotides and derivatives	40
Alkaloids	38
Terpenoids	21
Lignans and Coumarins	18
Tannins	4
Total	612

### Principal components analysis

**Fig. 2:**
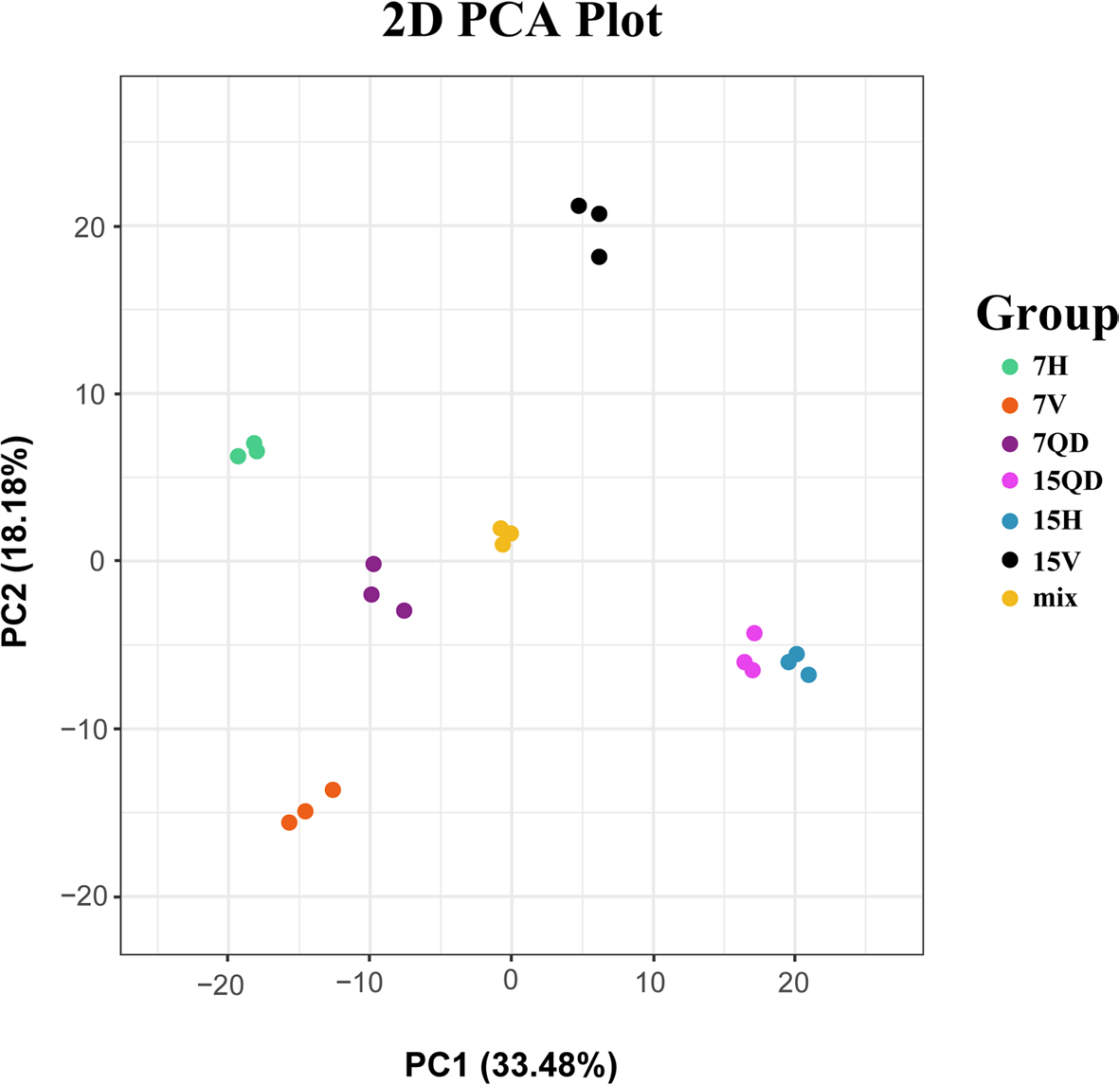
Principal component analysis (PCA) score map Each color represents one group. Mix means the data quality control group, inserting one mix every ten sample, three mix enriched together reflected the stability of device

Principal components analysis (PCA) shows CCYV infection greatly changed *C. sativus* metabolism, and different groups can separate from PC1 and PC2 (Fig. 2, PC1: 33.48%; PC2:18.18%). Additionally, samples at 7 dpi enriched at different positions from that at 15 dpi, indicating that CCYV changed the physiological traits of cucumber plants.

### Quantity statistics of significantly changed metabolites

**Fig. 3:**
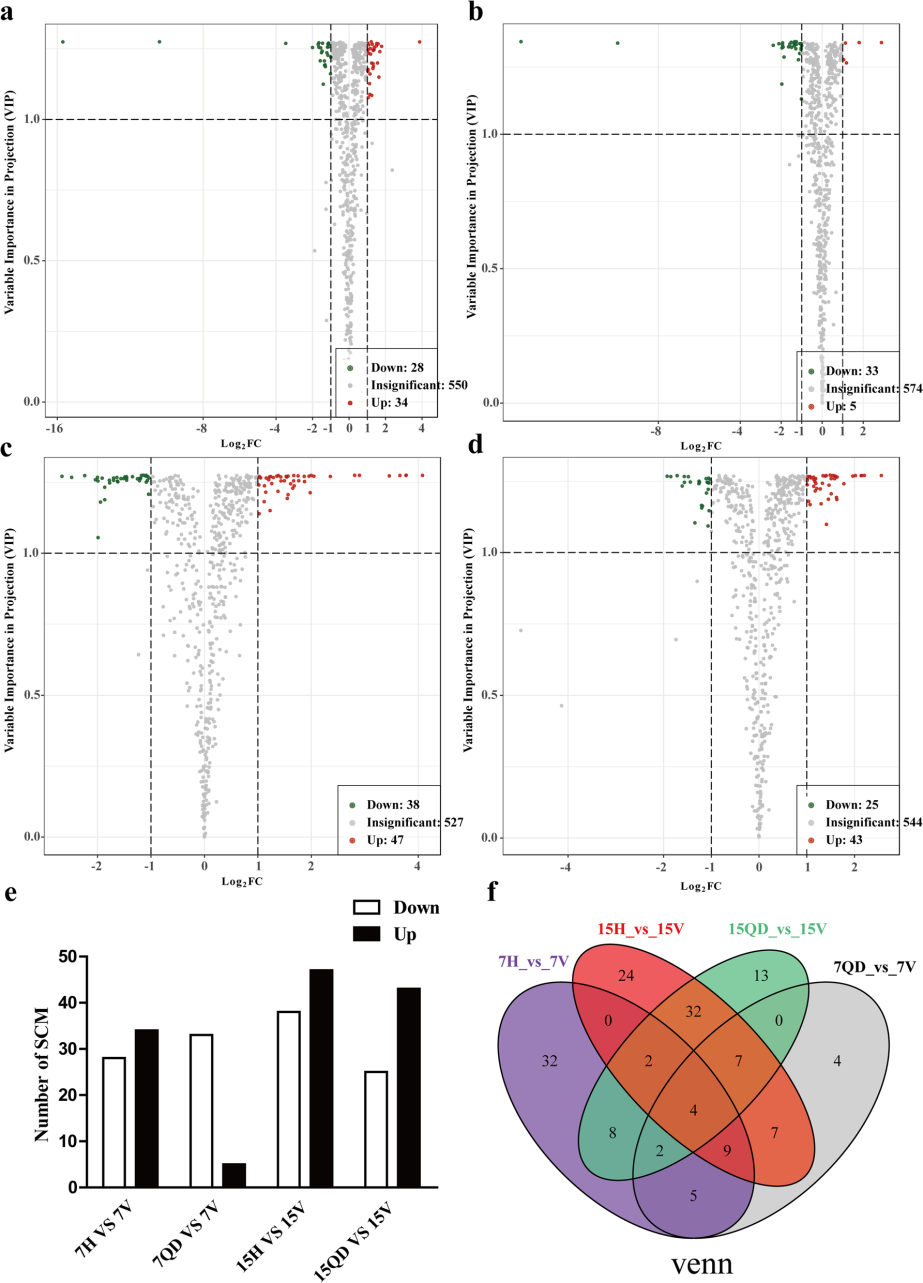
Quantity statistics of significantly changed metabolites. Volcanic plots (**a**): 7H VS 7 V, (**b**): 7QD VS 7 V, (**c**): 15H VS 15 V, (**d**): 15QD VS 15 V), The X-axis showed the Log_2_ FC = Log_2_ Fold Change, Y-axis showed the VIP value, each point represented one metabolite, the green point means the content of metabolites decreased (Log_2_ FC ≤ -1, VIP > 1), while the red point means the content of metabolites increased (Log_2_ FC ≥ 1, VIP > 1). Statistics of significantly changed metabolites (SCM) (**e**). Venn diagram (**f**). H represents healthy plants group, QD represents Q-biotype *Bemisia tabaci* damaged group, V represents CCYV-infected group

Volcano plot combined the Log_2_ FC and variable importance in project (VIP) value to screen differential metabolites (Fig. 3-a-d). Log_2_ FC ≥ 1, VIP ≥ 1 and Log_2_ FC ≤ -1, VIP ≥ 1 were selected. The screen results were shown in Table S2. In 7H VS 7 V comparison group, the content of 34 metabolites significantly increased and 28 metabolites decreased. In 7QD VS 7 V comparison group, a total of 34 metabolites significantly decreased while 5 metabolites notably increased. In 15H VS 15 V comparison group, a total of 47 metabolites significantly increased while 38 metabolites decreased, and in 15QD VS 15 V comparison group, there are 43 metabolites increased while 25 metabolites declined (Fig. 3e). The number of significantly changed metabolites increased with the CCYV infection period. There are four metabolites were commonly significantly changed in four comparison groups (Fig. 3f).

### Hierarchical cluster analysis (HCA)

**Fig. 4:**
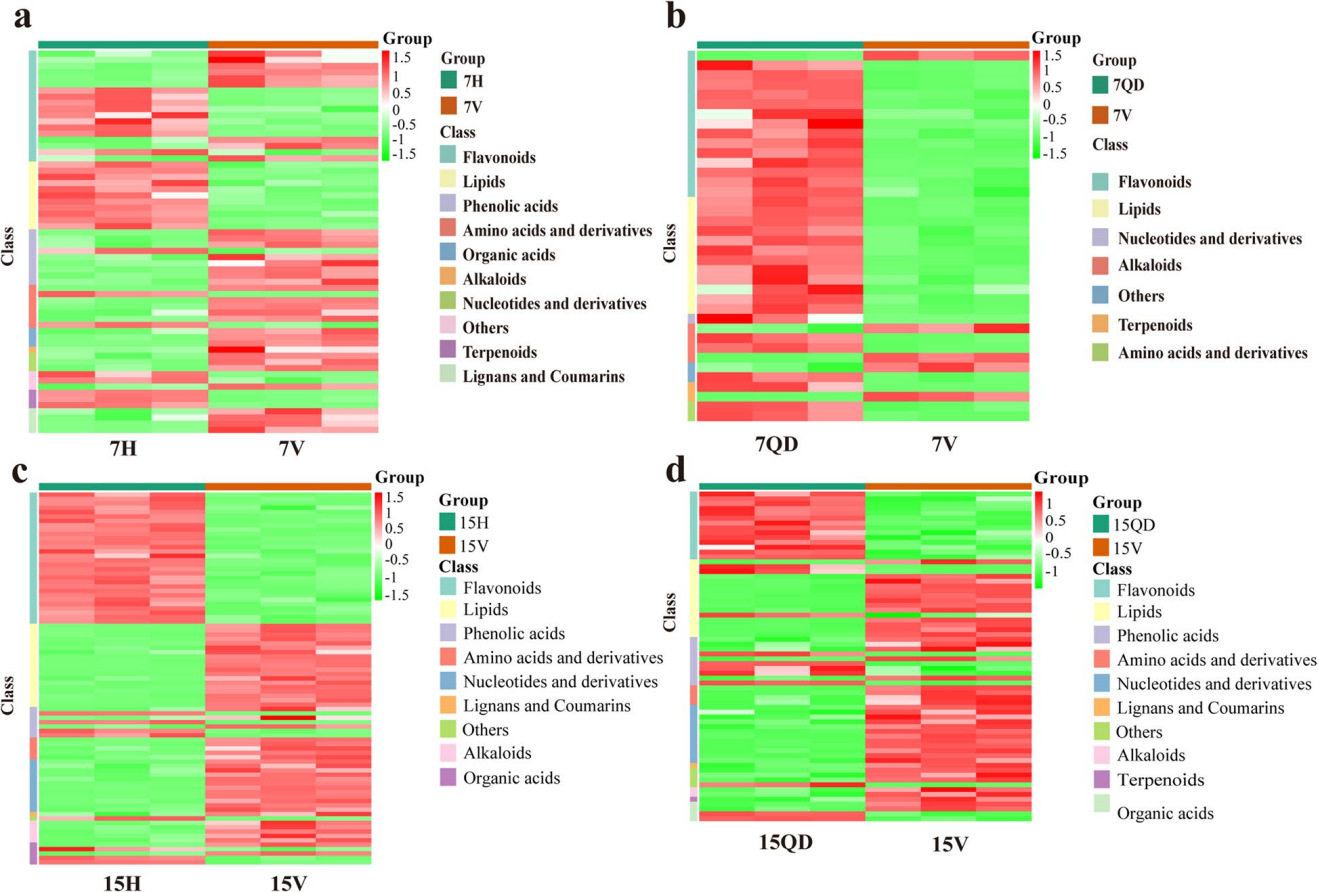
Hierarchical cluster analysis 7H VS 7 V (**a**), 7QD VS 7 V (**b**), 15H VS 15 V (**c**), 15QD VS 15 V (**d**). The ordinate shows the relative content of different class of metabolites. The green color indicates the low content, while the red color means high content. The information of metabolites can be referred in Table S1

The HCA can reflect the difference of content among metabolites by the color changes. The content increased with the color from green to red (Fig. 4). The information of metabolites can be found in Table S1. At 7 dpi, the content of flavonoids, lipids and terpenoids became lower in CCYV-infected plants, but the amino acids and their derivatives increased after CCYV infection. Additionally, there is a alkaloid, betaine, decreased after CCYV infection, while another alkaloid acetryptine up-accumulated. Two nucleotides and derivatives, lumazine and 2'-deoxyinosine-5'-monophosphate, presented high content in CCYV-infected cucumber plants. In 7H vs 7 V comparison group, there are nine phenolic acids and four organic acids increased, as well as a phenolic acid decreased after CCYV-infection. However, those difference did not appear in 7QD vs 7 V comparison group. At 15 dpi, the content of flavonoids decreased in CCYV-infected plants. More amino acids, nucleotides and their derivatives were detected and up-accumulated after CCYV-infection. Meanwhile, there are a organic acid, diethyl phosphate, and a phenolic acid, (S)-2-hydroxy-3-(4-hydroxyphenyl) propanoic acid, increased after CCYV infection. Interestingly, the content of lipids turned to be higher in CCYV-infected plants. Lipids and flavonoids took a big part in the significantly changed metabolites, while the terpenoids, organic acids, etc. only occupied a minor part.

### Functional annotation and differential metabolites enrichment analysis

**Fig. 5:**
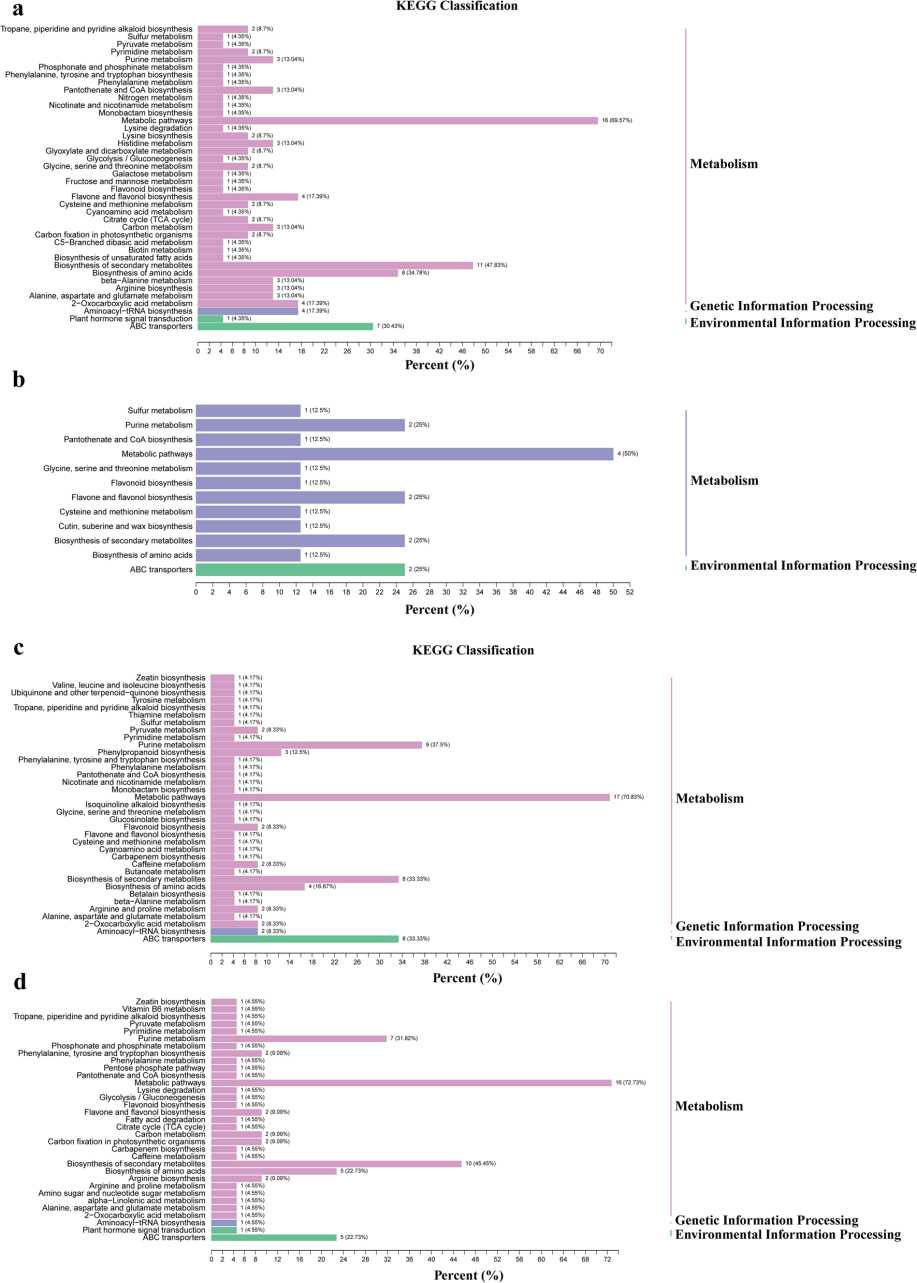
Differential metabolites enrichment analysis 7H VS 7 V (**a**), 7QD VS 7 V (**b**), 15H VS 15 V (**c**), 15QD VS 15 V (**d**). Different colors represented different kinds of metabolites, and the X-axis shows the percentage of different kinds of metabolites

The significantly changed metabolites were annotated by the Kyoto Encyclopedia of Genes and Genomes (KEGG) database. The enrichment analysis showed that differential metabolites mainly involve in biosynthesis of flavonoids, amino acids, alkaloids, etc. (Fig. 5). Additionally, some significantly changed metabolites were components of plant hormone transduction, ABC transporters and aminoacyl-tRNA biosynthesis.

### Screening of commonly changed metabolites

**Fig. 6:**
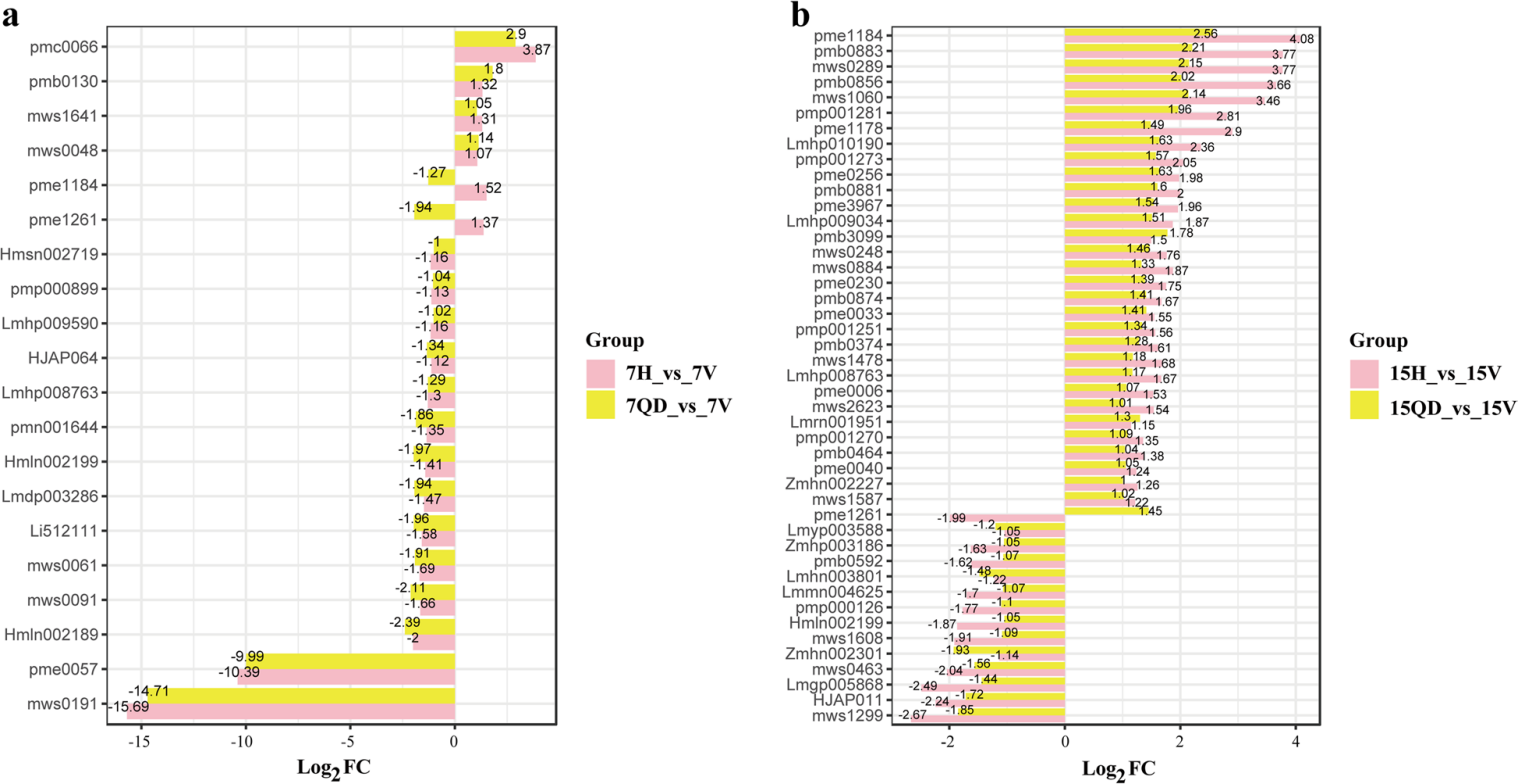
Screening of commonly changed metabolites 7H VS 7 V & 7QD VS 7 V (**a**), 15H VS 15 V & 15QD VS 15 V (**b**). Log_2_ FC = Log_2_ (fold change), Log_2_ FC > 0 indicates that the content of metabolites increased after CCYV infection, Log_2_ FC < 0 means the content decreased

At 7 dpi, a total of 16 commonly changed metabolites (changed in both comparison group with the same tendency) were detected, including 10 flavonoids, 2 lipids and other kinds of metabolites (Fig. 6, Table 2, Table S3). Both the commonly differential flavonoids and lipids decreased. At 15 dpi, there were 43 commonly changed metabolites, including 11 flavonoids, 13 lipids and 10 nucleotides and their derivatives, 2 amino acids and derivatives, 2 alkaloids, and 4 phenolic acids. Most flavonoids commonly changed in each comparison group showed lower content in CCYV infection group. Interestingly, the commonly changed lipids mainly are LPE (lysophosphatidylethanolamine), LPC (lysophosphatidylcholine). The HCA showed that lipids decreased at 7 dpi but significantly increased at 15 dpi (Fig. 4). Thus, the lipids may play vital roles in CCYV and *C. sativus* interactions.

**Table 2:** Quantity statistical analysis of commonly changed metabolites

Class		7 dpi (Number of metabolites)	15 dpi (Number of metabolites)
Flavonoids	down	8	11
	up	2	0
Lipids	down	2	0
	up	0	13
Amino acids and derivatives	down	1	0
	up	0	3
Nucleotides and derivatives	down	0	0
	up	1	10
Alkaloids	down	1	0
	up	1	2
Phenolic acids	down	0	2
	up	0	2

## Discussion

Plants are able to defend themselves against herbivore insects by producing various secondary metabolites, like flavonoids and tannins. Those chemicals are difficult to digest or are poisonous to insects [24, 25]. Lipids are one of the major components of biological membranes such as the plasma membrane and membranes of organelles, and involve in movement of signaling molecules [26]. Amino acids and derivatives have been revealed that involving in plant response to various biotic and abiotic stresses [27, 28]. Pérez-Clemente et al. [29] have reported that Citrus tristeza virus (CTV) infection induced accumulation of amino acids and derivatives. Here, we also found the high accumulation of amino acids and their derivatives in CCYV-infected plants (Fig. 4). The increase of amino acids within plants is conducive for the growth and development of herbivore insects [14, 30], which means that CCYV infection enhanced the fitness of cucumber plants to whitefly. The reduction of flavonoids and terpenoids after CCYV infection also means a positive change for whitefly feeding on CCYV-infected cucumber plants. Flavonoids and terpenoids affect feeding behaviors and are harmful to the growth and development of herbivore insects [31–33]. Meanwhile, terpenoids in plant volatile are crucial cues for natural enemy locating their hosts [34, 35]. Viruses protect their insect vectors have been reported previously. Luan et al. (2013) proved that Tomato yellow leaf curl China virus infection depleted the terpenoid-mediated plant defence against whiteflies to promote its mutualism with vector whiteflies [32]. CCYV-induced the reduction of terpenoids in cucumber plants would possibly protect vector whiteflies. Our previous studies indicated that CCYV infection made the host plant *C. sativus* susceptible to vector whitefly, and whiteflies showed better performance on CCYV-infected cucumber plants [21, 22]. Therefore, these changes are consistent with our speculation that CCYV modified the physiology of host plant, and thus improving whiteflies feeding on CCYV-infected host plants. More importantly, this change seems favorable for CCYV transmission.

Additionally, we found that the content of lipids reduced in CCYV-infected plants at 7 dpi but increased at 15 dpi. This transition implied that lipids play vital roles in CCYV infecting cucumber plant successfully. Not only the lipids can be employed by plants to resist against the infection of viruses [36] but the lipids metabolize pathways can be hijacked by plant viruses. Zhang et al. [37] reported that the enrichment of lipids is correlated with the recruitment of the necessary components for virus replication, such as RNA-dependent RNA polymerase (RdRP). In this study, the differential lipids mainly are LPE and LPC, which are the degradation products of acetylphosphatidylcholine (PC) and acetylphosphatidylethanolamine (PE). Xu et al. [38] found that LPE and PE can improve tomato bushy stunt virus (TBSV) replication by promoting the formation of viral replicase complex (VRC) assembly. LPC was identified to influence membrane fusion and fission, and the movement of some animal viruses within organisms, like TBEV (tick-borne encephalitis virus) [39–41]. Therefore, we speculated that CCYV may hijack lipids metabolism of host plant for replication and infection. Furthermore, at both 7 and 15 dpi, the content of nucleotides and derivatives increased upon CCYV-infection and the number of changed nucleotides also increased with the infection period. The changed adenine and guanine have been proved that utilizing for RNA synthesis. CCYV is a single strain RNA virus with a bipartite genome [42]. Our recent work proved that the titers of CCYV within host plant increased from 10 to 30 dpi [22]. Therefore, the up-accumulation of nucleotides and lipids possibly provide raw materials and sites for CCYV replication and assembly.

## Conclusions

Here, we found CCYV infection repressed the flavonoids and terpenoids related defense responses of cucumber plants but triggered lipids, nucleotides and amino acids metabolize. These changes would enhance the fitness of cucumber plants to whiteflies and help CCYV establish infection within cucumber plants. This study provided an insight to help understand how cucumber plants respond to CCYV infection at different inoculation periods. Importantly, it provided information to seed breeding for pest management and plant viral diseases control.

## Material and methods

To obtain a set of CCYV-infected host plants, viruliferous *B. tabaci* Q biotype (Mediterranean, MED) feeding on the host plant for three days (acquisition-access period, AAP) to get CCYV-infected host plants marked with ‘V’. In order to eliminate the influence of insect feeding, we set plants with non-viruliferous *B. tabaci* feeding for three days marked with Q-biotype damage (QD), together with healthy (H) plants are used as controls. Samples were collected at 7 and 15 dpi.

### Plant material and insect vector

The cucumber seeds were obtained commercially from Tianjin Derit Seeds Company Ltd (Tianjin, China). Cucumber plants (*C. sativus*, var. Bojie-107) were cultivated in the plastic pots (Diameter = 10 cm, Height = 12 cm), and maintained in a greenhouse with photoperiod Light: Dark = 16: 8, temperature 27 ± 3 ℃, relative humidity: 40 ± 5%. Seedlings with 3—4 true leaves were used for experiments.

Viruliferous *B. tabaci* Q biotype as a vector to inoculate the host plant with CCYV. The colony of *B. tabaci* was maintained on healthy cucumber plants. The genetic purity of *B. tabaci* Q biotype was monitored according to Li et al. [17]. Transferring non-viruliferous *B. tabaci* adults onto CCYV-infected cucumber plants for a 3-d acquisition-access period (AAP). Then, fifty pairs of virus-free and viruliferous female and male whitefly adults were separately transferred into the clip cages and kept on the leaves of healthy plants for three days for inoculation [43], and then the whitefly (including eggs, nymphs and adults) were removed from plants.

### Identification of CCYV-infected plants

Plant leaves or whiteflies were ground into powder in liquid nitrogen. The total RNA was extracted with Trizol (Takara Bio, Shiga, Japan) based on the manufacturer’s instructions. Total RNA (1 μg) was used with PrimerScript RT reagent kit (Takara) for reverse transcription according to the instructions. CCYV-infected plants and viruliferous whiteflies were identified by RT-PCR [44, 45].

Reactions were conducted in a total volume of 20 μl containing 10 μL Premix Taq™ (Ex Taq™ Version 2.0 plus dye, Takara), 0.5 μL (5 μM) of each primer, 1μL cDNA sample and 8μL ddH_2_O. Amplification reactions were performed as follows: 94℃ for 2 min; 35 cycles of 94℃ for 30 s, 55 ℃ for 30 s and 72℃ for 30 s. PCR products (8 μL) were subjected to electrophoresis in a 1.2% agarose gel 1 × TAE buffer (40 mM Tris–acetate, pH 8.3, 1 mM EDTA), and observed agarose gels on a UV-transilluminator.

### Sample preparation and extraction

**Fig. 7:**
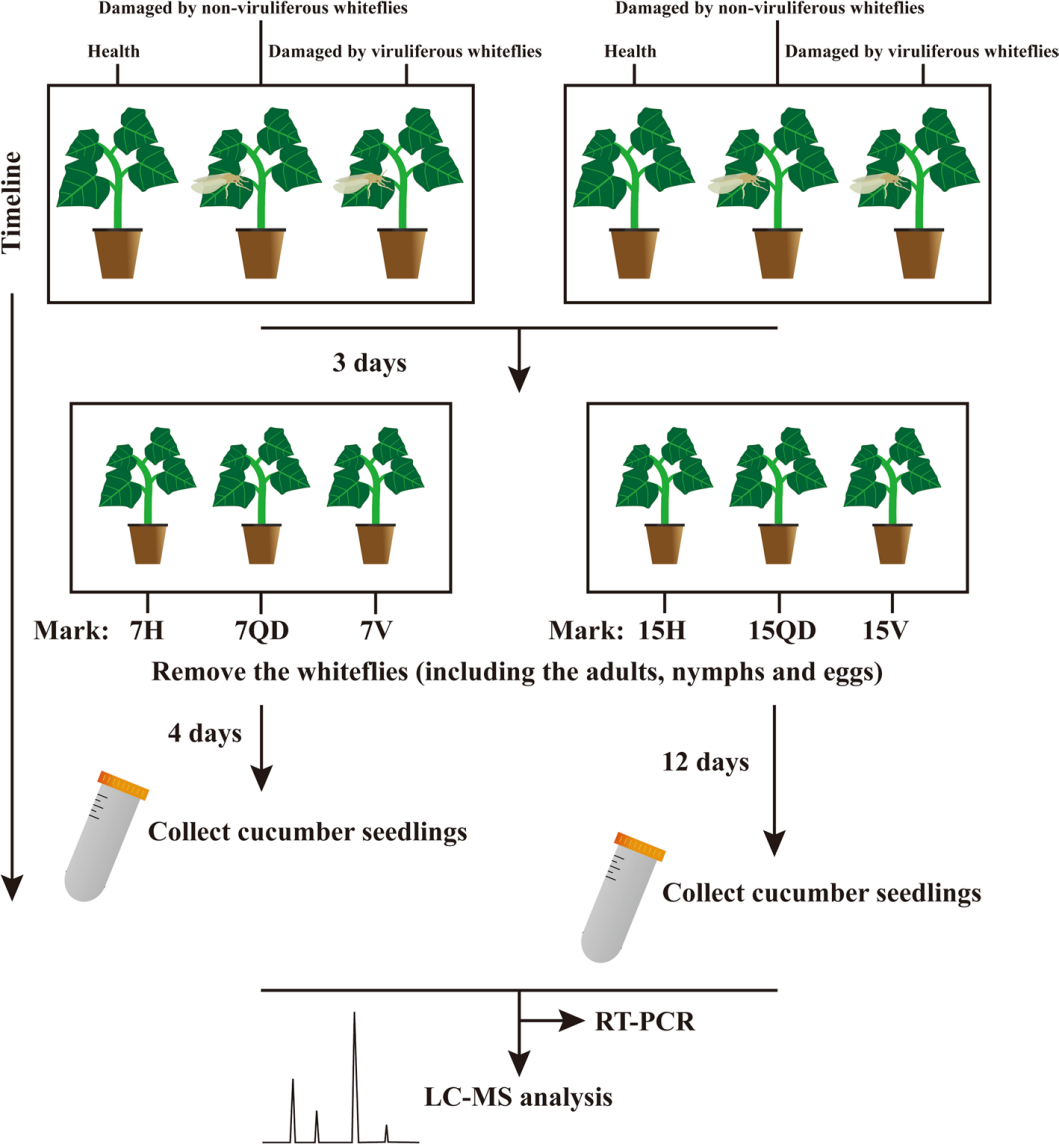
Experimental design and work flow of this study H: healthy plants, QD: Q-biotype whiteflies damaged plants, V: CCYV-infected plants

Samples were collected at 7 and 15 dpi with liquid nitrogen (Fig. 7). One hundred mg freeze-dried sample was crushed using a mixer mill (MM 400, Retsch) with a zirconia bead for 1.5 min at 30 Hz. The powder was extracted overnight at 4 °C with 1.2 mL 70% aqueous methanol. Following centrifugation at 12000 rpm for 10 min, the extracts were filtrated (SCAA-104, 0.22 μm pore size; ANPEL, Shanghai, China) before UPLC-MS/MS analysis.

### UPLC Conditions

Extracts were analyzed using an UPLC-ESI–MS/MS system (UPLC, SHIMADZU Nexera X2, www. shimadzu. com. cn/; MS, Applied Biosystems 4500 Q TRAP, www. appliedbiosystems. com. cn/). The analytical conditions were shown as follows, UPLC: column, Agilent SB-C18 (1.8 µm, 2.1 mm*100 mm); The mobile phase was consisted of solvent A, pure water with 0.1% formic acid, and solvent B, acetonitrile with 0.1% formic acid. Sample measurements were performed with a gradient program that employed the starting conditions of 95% A, 5% B. Within 9 min, a linear gradient to 5% A, 95% B was programmed, and a composition of 5% A, 95% B was kept for 1 min. Subsequently, a composition of 95% A, 5.0% B was adjusted within 1.10 min and kept for 2.9 min. The column oven was set to 40 °C; The injection volume was 4 μL. The effluent was alternatively connected to an ESI-triple quadrupole-linear ion trap (QTRAP)-MS.

### ESI-Q TRAP-MS/MS

LIT and triple quadrupole (QQQ) scans were acquired on a triple quadrupole-linear ion trap mass spectrometer (Q TRAP), AB4500 Q TRAP UPLC/MS/MS System, equipped with an ESI Turbo Ion-Spray interface, operating in positive and negative ion mode and controlled by Analyst 1.6.3 software (AB Sciex). The ESI source operation parameters were as follows: ion source, turbo spray; source temperature 550 °C; ion spray voltage (IS) 5500 V (positive ion mode)/-4500 V (negative ion mode); ion source gas I (GSI), gas II (GSII), curtain gas (CUR) were set at 50, 60, and 25.0 psi, respectively; the collision gas (CAD) was high [46]. Instrument tuning and mass calibration were performed with 10 and 100 μmol/L polypropylene glycol solutions in QQQ and LIT modes, respectively. QQQ scans were acquired as MRM experiments with collision gas (nitrogen) set to medium. DP and CE for individual MRM transitions was done with further DP and CE optimization. A specific set of MRM transitions were monitored for each period according to the metabolites eluted within this period [47].

### Data analysis

#### PCA analysis

Unsupervised PCA (principal component analysis) was performed by statistics function prcomp within R. The data was unit variance scaled before unsupervised PCA.

#### Hierarchical cluster analysis and pearson correlation coefficients

The hierarchical cluster analysis (HCA) results of samples and metabolites were presented as heatmaps with dendrograms, while *Pearson correlation coefficients* (PCC) between samples were calculated by the cor function in R and presented as only heatmaps. Both HCA and PCC were carried out by R package pheatmap. For HCA, normalized signal intensities of metabolites (unit variance scaling) were visualized as a color spectrum.

#### Comparision of altered metabolites in plants inoculated with or without virus

Significantly regulated metabolites between groups were determined by VIP ≥ 1 and absolute Log_2_FC (fold change) ≥ 1. VIP values were extracted from OPLS-DA result, which also contain score plots and permutation plots, was generated using R package MetaboAnalystR. The data was log transform (log2) and mean centering before OPLS-DA. In order to avoid overfitting, a permutation test (200 permutations) was performed.

#### KEGG annotation and enrichment analysis

Identified metabolites were annotated using KEGG Compound database (http://www.kegg.jp/kegg/compound/) [48], annotated metabolites were then mapped to KEGG Pathway database (http://www.kegg.jp/kegg/pathway.html). Pathways with significantly regulated metabolites mapped to were then fed into MSEA (metabolite sets enrichment analysis), their significance was determined by hypergeometric test’s *p*-values.

## Supplementary Information

**Additional file 1: Table S1. **All metabolites detected in metabolomic analysis.

**Additional file 2: Table S2. **All differential metabolites in leaves of cucumber plants in each group.

**Additional file 3: Table S3. **Information of commonlychanged metabolites in each comparison group.

**Additional file 4: Figure S1. **Identification of cucumberplants and whiteflies infected by CCYV.

**Additional file 5: Figure S2.** Identification of non-viruliferouswhiteflies.

## Data Availability

All data generated or analyzed during this study are included in this published article.
