# Supplementary figures and images for: Widely targeted analysis of metabolomic changes of Cucumis sativus induced by cucurbit chlorotic yellows virus
Source: BMC Plant Biol. 2022 Mar 31;22:158. doi: 10.1186/s12870-022-03555-3 (PMC8969345; doi:10.1186/s12870-022-03555-3)

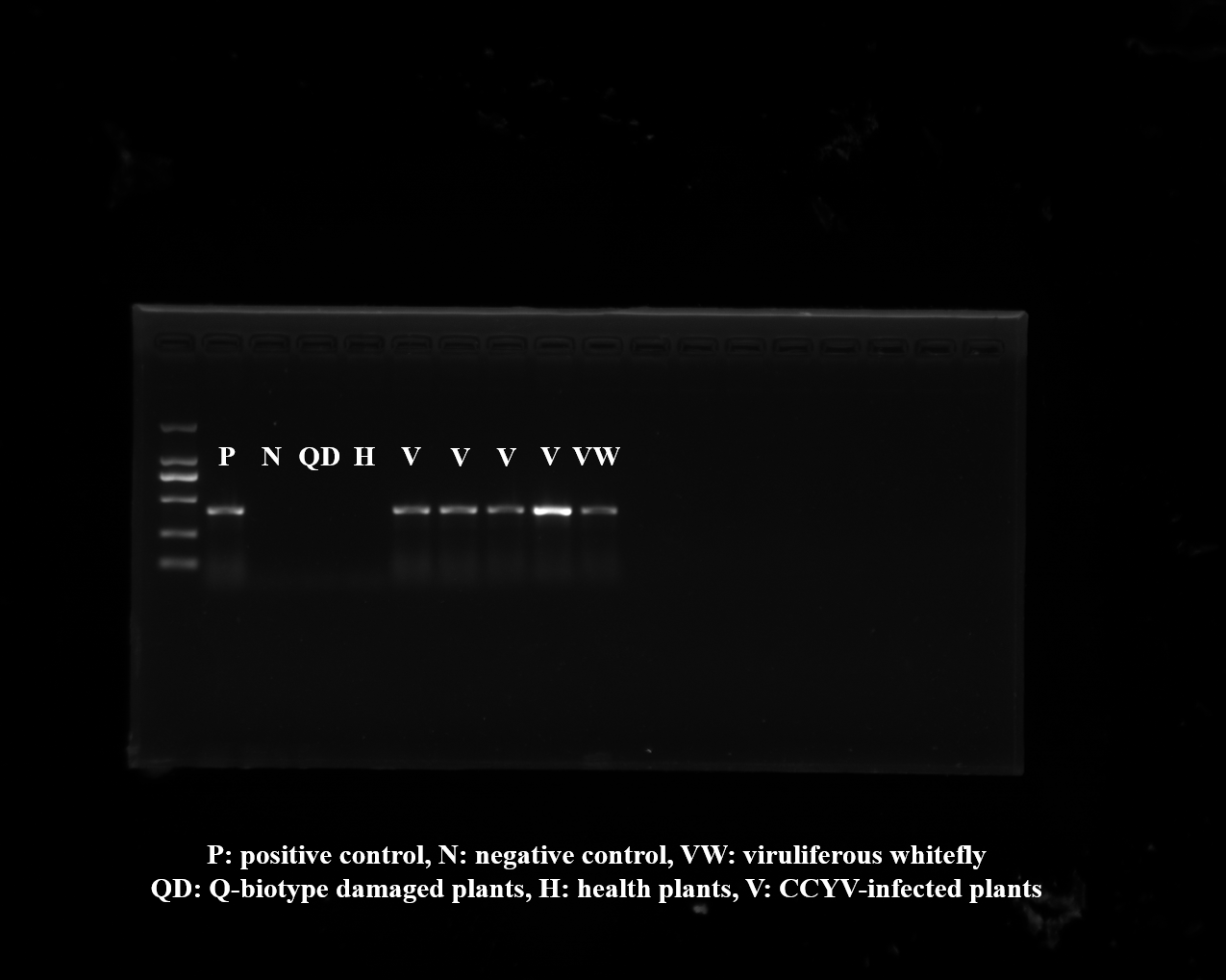

Supplement: Supplementary file 4 — Additional file 4: Figure S1. Identification of cucumberplants and whiteflies infected by CCYV. [file 12870_2022_3555_MOESM4_ESM.tif]

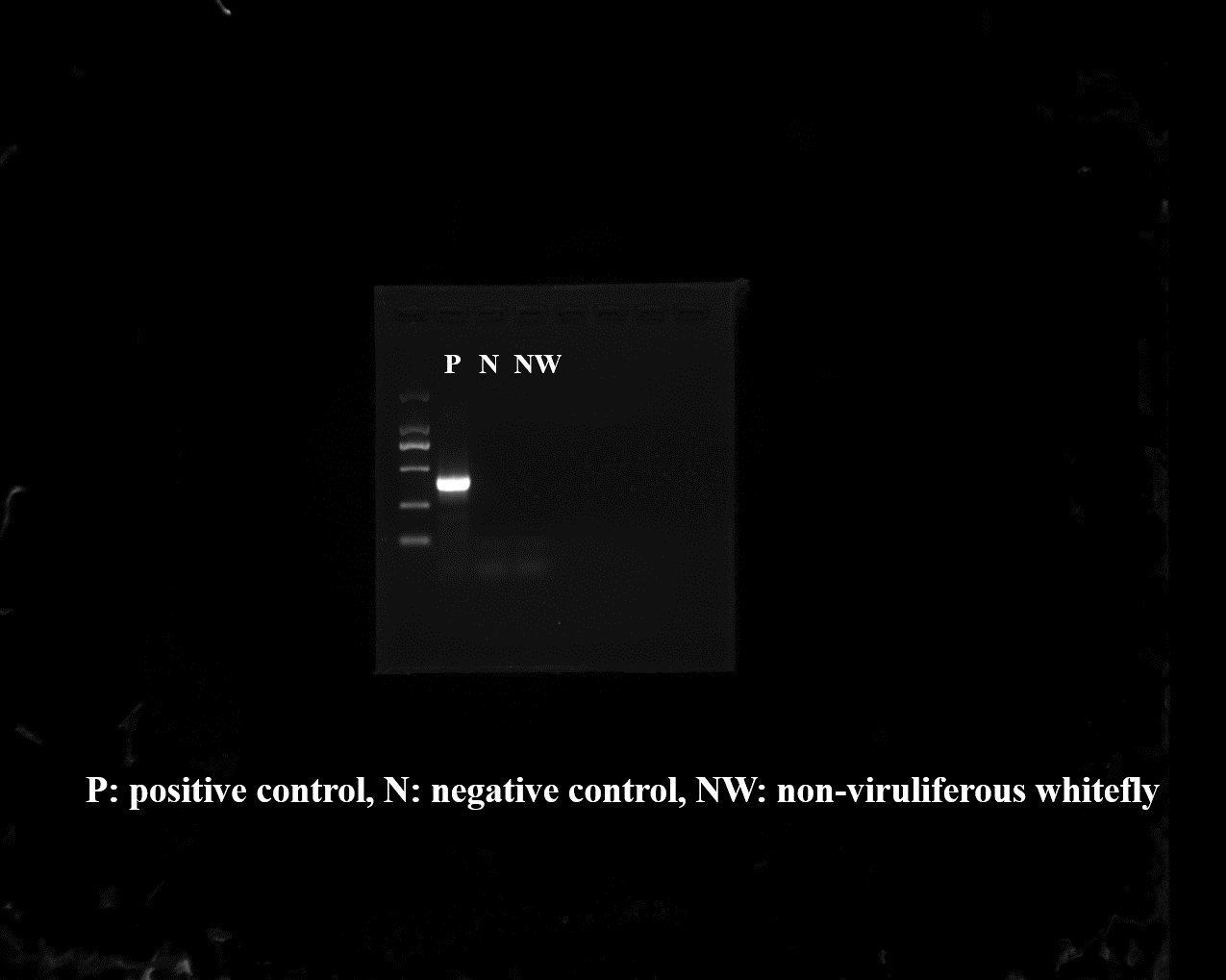

Supplement: Supplementary file 5 — Additional file 5: Figure S2. Identification of non-viruliferouswhiteflies. [file 12870_2022_3555_MOESM5_ESM.png]
